# Supplementary material for: Robust measurement of microbial reduction of graphene oxide nanoparticles using image analysis
Source: Appl Environ Microbiol. 2025 Mar 27;91(4):e00360-25. doi: 10.1128/aem.00360-25 (PMC12016504; doi:10.1128/aem.00360-25)
Supplement: Supplemental material — Tables for media preparation, user image selection, and code. [file aem.00360-25-s0001.docx]

**Supplemental Table 1: Individual p-value comparisons using an unpaired t-test for each user selection**

|  | User 1 | User 2 | User 3 | User 4 | User 5 | User 6 |
| --- | --- | --- | --- | --- | --- | --- |
| User 1 |  | 0.8928 (3.323, 26) | **0.0132 (2.667,25)** | **0.0002 (4.527,23)** | **0.0004 (3.949,36)** | **0.0088 (2.840,25)** |
| User 2 | 0.8928 (3.323, 26) |  | **0.0102 (3.323,26)** | **<0.0001 (6.095,18)** | **0.0014 (3.502,31)** | **0.0101 (2.839,20)** |
| User 3 | **0.0132 (2.667,25)** | **0.0102 (3.323,26)** |  | 0.1049 (1.699,20) | **<0.0001 (5.330,33)** | **0.0001 (4.676,22)** |
| User 4 | **0.0002 (4.527,23)** | **<0.0001 (6.095,18)** | 0.1049 (1.699,20) |  | **<0.0001 (5.998,31)** | **<0.0001 (5.929,20)** |
| User 5 | **0.0004 (3.949,36)** | **0.0014 (3.502,31)** | **<0.0001 (5.330,33)** | **<0.0001 (5.998,31)** |  | 0.2389 (1.199,33) |
| User 6 | **0.0088 (2.840,25)** | **0.0101 (2.839,20)** | **0.0001 (4.676,22)** | **<0.0001 (5.929,20)** | 0.2389 (1.199,33) |  |

**Supplemental Table 2: Unpaired T-test comparison of p-values for plate reader analysis**

|  | Control | O.D._600_ 0.02 | O.D._600_ 0.05 | O.D._600_ 0.10 | O.D._600_ 0.16 | O.D._600_ 0.3 |
| --- | --- | --- | --- | --- | --- | --- |
| Control |  | **0.0017**  **(3.773,16)** | **0.0015**  **(3.827,16)** | **0.0043**  **(3.403,14)** | **0.0049**  **(3.332,14)** | **0.0011**  **(4.112,14)** |
| O.D._600_ 0.02 | **0.0017**  **(3.773,16)** |  | 0.5481  (0.6136,16) | 0.3881 (0.8907,14) | 0.2991 (1.078,14) | **0.0493 (2.153,14)** |
| O.D._600_ 0.05 | **0.0015**  **(3.827,16)** | 0.5481  (0.6136,16) |  | 0.7185  (0.3678,14) | 0.5694  (0.5826,14) | 0.1170  (1.670,14) |
| O.D._600_ 0.10 | **0.0043**  **(3.403,14)** | 0.3881 (0.8907,14) | 0.7185  (0.3678,14) |  | 0.8467  (0.1976,12) | 0.2750  (1.144,12) |
| O.D._600_ 0.16 | **0.0049**  **(3.332,14**) | 0.2991 (1.078,14) | 0.5694  (0.5826,14) | 0.8467  (0.1976,12) |  | 0.3722  (0.9271,12) |
| O.D._600_ 0.3 | **0.0011**  **(4.112,14)** | **0.0493 (2.153,14)** | 0.1170  (1.670,14) | 0.2750  (1.144,12) | 0.3722  (0.9271,12) |  |

**Supplemental Table 3: Unpaired T-test comparison of p-values for image analysis**

|  | Control | O.D._600_ 0.02 | O.D._600_ 0.05 | O.D._600_ 0.10 | O.D._600_ 0.16 | O.D._600_ 0.3 |
| --- | --- | --- | --- | --- | --- | --- |
| Control |  | **0.0003**  **(4.753,14)** | **0.0005**  **(4.471,14)** | **0.0033**  **(3.659,12)** | **0.0018**  **(3.998,12)** | **0.0007**  **(4.543,12)** |
| O.D._600_ 0.02 | **0.0003**  **(4.753,14)** |  | 0.8550  (0.1857) | 0.6837  (0.4161,14) | 0.8307  (0.2178,14) | 0.4491  (0.7787,14) |
| O.D._600_ 0.05 | **0.0005**  **(4.471,14)** | 0.8550  (0.1857) |  | 0.5877  (0.5549,14) | 0.9594  (0.0518,14) | 0.5668  (0.5867,14) |
| O.D._600_ 0.10 | **0.0033**  **(3.659,12)** | 0.6837  (0.4161,14) | 0.5877  (0.5549,14) |  | 0.6029  (0.5343,12) | 0.3279  (1.020,12) |
| O.D._600_ 0.16 | **0.0018**  **(3.998,12)** | 0.8307  (0.2178,14) | 0.9594  (0.0518,14) | 0.6029  (0.5343,12) |  | 0.6472  (0.4694,12) |
| O.D._600_ 0.3 | **0.0007**  **(4.543,12)** | 0.4491  (0.7787,14) | 0.5668  (0.5867,14) | 0.3279  (1.020,12) | 0.6472  (0.4694,12) |  |

**Minimal media composition**

To prepare Minimal media, components for Minimal media (Supplemental Table 4), vitamin stock (Supplemental Table 5), mineral stock (Supplemental Table 6), or amino acid stock (Supplemental Table 7) were brought to pH 7 using HCl or NaOH solution. All solutions were autoclaved for 30 minutes at 121 ºC. All solutions were then combined in the ratios listed in Table 1. Stock solutions were frozen in 5 mL aliquots at -4 ºC to avoid multiple freeze-thaw cycles.

**Supplemental Table 4: Minimal media components**

| **Chemical name** | **g / L** | **Formula** | **Vendor** | **Final concentration in minimal media (mM)** |
| --- | --- | --- | --- | --- |
| PIPES buffer | 15.16 | C_8_H_18_N_2_O_6_S_2_ | Sigma-Aldrich | 50 |
| Sodium hydroxide | 0.3 | NaOH | Sigma-Aldrich | 7.5 |
| Ammonium chloride | 1.5 | NH_4_Cl | Sigma-Aldrich | 28.04 |
| Potassium chloride | 0.1 | KCl | Sigma-Aldrich | 28.04 |
| Sodium phosphate monobasic | 0.6 | NaH_2_PO_4_·H_2_O | Sigma-Aldrich | 4.35 |
| Sodium chloride | 1.75 | NaCl | Fisher Scientific | 30 |
| Vitamins solution 10000x stock | 100 µL |  |  | 1x |
| Mineral solution 100x stock | 10 mL |  |  | 1x |
| Amino acid solution 100x stock | 10 mL |  |  | 1x |

**Supplemental Table 5: Vitamin solution 10000x stock**

| **Chemical name** | **g / 100 mL** | **Formula** | **Vendor** | **Final concentration in minimal media (nM)** |
| --- | --- | --- | --- | --- |
| Biotin (d-biotin) | 0.02 | C_10_H_16_N_2_O_3_S | Sigma-Aldrich | 81.87 |
| Folic acid | 0.02 | C_19_H_19_N_7_O_6_ | Sigma-Aldrich | 45.34 |
| Pyridoxal HCl | 0.1 | C_8_H_12_ClNO_3_ | Sigma-Aldrich | 486.38 |
| Riboflavin | 0.05 | C_17_H_20_N_4_O_6_ | Sigma-Aldrich | 132.84 |
| Thiamine HCl | 0.05 | C_18_H_18_Cl_2_N_4_OS | Sigma-Aldrich | 140.73 |
| Nicotinic acid | 0.05 | C_6_H_5_NO_2_ | Sigma-Aldrich | 406.17 |
| D-pantothenic acid, hemicalcium salt | 0.05 | C_9_H_16_NO_5_·$\frac{1}{2}$Ca | Sigma-Aldrich | 209.82 |
| Vitamin B12 | 0.0001 | C_63_H_88_CoN_14_O_14_P | Sigma-Aldrich | 0.74 |
| 4-aminobenzoic acid | 0.05 | C_7_H_7_NO_2_ | Sigma-Aldrich | 364.62 |
| Thioctic acid (α-lipoic acid) | 0.05 | C_8_H_14_O_2_S_2_ | Sigma-Aldrich | 242.37 |

**Supplemental Table 6: Mineral solution 100x stock**

| **Chemical name** | **g / 100 mL** | **Formula** | **Vendor** | **Final concentration in minimal media (µM)** |
| --- | --- | --- | --- | --- |
| Nitrilotriacetic acid (bring to pH 8 with NaOH) | 1.5 | C_6_H_9_NO_3_ | Sigma-Aldrich | 78.49 |
| Magnesium sulfate heptahydrate | 3 | MgSO_4_·7H_2_O | Sigma-Aldrich | 121.71 |
| Manganese sulfate monohydrate | 0.5 | MnSO_4_·H_2_O | Sigma-Aldrich | 29.58 |
| Sodium Chloride | 1 | NaCl | Sigma-Aldrich | 171.12 |
| Ferrous sulfate heptahydrate | 0.1 | FeSO_4_·7H_2_O | Sigma-Aldrich | 3.60 |
| Calcium chloride dihydrate | 0.1 | CaCl_2_·2H_2_O | Sigma-Aldrich | 6.80 |
| Cobalt chloride hexahydrate | 0.12 | CoCl_2_·6H_2_O | Sigma-Aldrich | 5.20 |
| Zinc chloride | 0.13 | ZnCl_2_ | Sigma-Aldrich | 9.54 |
| Cupric sulfate pentahydrate | 0.01 | CuSO_4_·5H_2_O | Sigma-Aldrich | 0.40 |
| Aluminum potassium disulfate dodecahydrate | 0.01 | AlK(SO_4_)_2_·12H_2_O | Sigma-Aldrich | 0.21 |
| Boric acid | 0.01 | H_3_BO_3_ | Sigma-Aldrich | 1.62 |
| Sodium molybdate dihydrate | 0.025 | Na_2_MoO_4_·2H_2_O | Sigma-Aldrich | 1.03 |
| Nickel chloride hexahydrate | 0.024 | NiCl_2_·6H_2_O | Sigma-Aldrich | 1.01 |
| Sodium tungstate | 0.025 | Na_2_WO_4_·2H_2_O | Sigma-Aldrich | 0.76 |

**Supplemental Table 7: Amino acid solution 100x stock**

| **Chemical name** | **g / L** | **Formula** | **Vendor** | **Final concentration in minimal media (mg/ mL)** |
| --- | --- | --- | --- | --- |
| L-glutamic acid | 0.2 |  | Sigma-Aldrich | 2 |
| L-arginine | 0.2 |  | Sigma-Aldrich | 2 |
| D-or L-serine | 0.2 |  | Sigma-Aldrich | 2 |

**Image Analysis Code**

%% Code authored by Danielle Bennett in MATLAB^TM^, last edit 11.18.2024. dbenne15@ur.rochester.edu

%% Code written to obtain average hue values for graphene oxide samples.

% A region of interest is first selected containing the graphene oxide to be selected. Then

% the selection is cropped. User then clicks points which do not contain imaging

% abberations (at least 15) and double click to process. The code

% selects a 32 x 32 pixel box around each selected point, averages the vector values and

% outputs the average and standard deviation.

%% Inputs: image file name in format "image.jpg"

%% Outputs: vector "results" containing the average value, standard deviation of all points, and number of points selected.

%Load image

11. im=imread("0hr_1d.jpg");

% Select rectangle on image which contains region of interest, and crop.

13. figure()

14. imshow(im)

15. [J,rect] = imcrop(im);

16. figure()

17. imshow(J)

%% Double click when points are done

19. [x,y] = getpts; % Select points to draw a 32 x 32 pixel box around (or whatever size)

20.

%Average points to get hue average

22.

23. [num_points,dim]=size(x);

24. sizebox=32; % This is the size of the box in pixels

25. hsv_J=rgb2hsv(J);%rgb2hsv(J);

%hue_av=zeros(num_points,1); % uncomment for other vector values

%sat_av=zeros(num_points,1);

28. val_av=zeros(num_points,1);

29. for i=1:num_points % get the average of each vector for the pixel box selected and return as list.

30. xdim=x(i)-sizebox/2;

31. ydim=y(i)-sizebox/2;

32. lil_image=imcrop(hsv_J,[xdim ydim sizebox sizebox]);

%hue_av(i)=mean(lil_image(:,:,1),'all');

%sat_av(i)=mean(lil_image(:,:,2),'all');

35. val_av(i)=mean(lil_image(:,:,3),'all');

36.

37. end

38.

%HSVAV=[hue_av sat_av val_av];

40. av=mean(HSVAV);

41. S=std(HSVAV);

42. results=[av(:,3) S(:,3) size(HSVAV,1)]; % the output vector

43.

%%

45.

**Supplemental Figures**

Supplemental Figure 1. Standard error as a percentage of average value measurement with varying number of sample points.

Supplemental Figure 2: Comparison of all three components of the HSV color space changing with respect to varying graphene oxide concentration. The average value of the hue, saturation, and value vectors are shown at a range of graphene oxide concentrations. The errors represent the standard deviation of the average values.

**
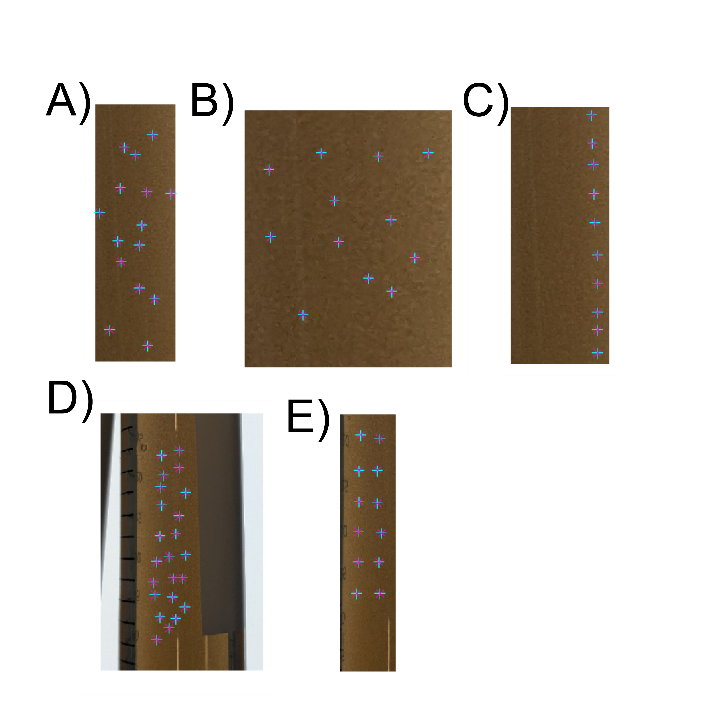
**

Supplemental Figure 3: User selections of points from the same image of microbial graphene oxide reduction reactions containing 0.05 % graphene oxide at the 3.5-hour timepoint for A) user 1, B) user 2, C) user 3, D) user 4, and E) user 5.

Supplemental figure 4: All datapoints for main text figure 4.

Supplemental figure 5: All datapoints for main text figure 5.

Supplemental figure 6: All datapoints for main text figure 6.
